# Clinical feasibility of Ethos auto-segmentation for adaptive whole-breast cancer treatment

**DOI:** 10.3389/fonc.2024.1507806

**Published:** 2024-12-10

**Authors:** Jessica Prunaretty, Fatima Mekki, Pierre-Ivan Laurent, Aurelie Morel, Pauline Hinault, Celine Bourgier, David Azria, Pascal Fenoglietto

**Affiliations:** Radiotherapy Department, Montpellier Regional Cancer Institute, Montpellier, France

**Keywords:** Ethos, auto-segmentation, artificial intelligence, breast cancer, adaptive treatment

## Abstract

**Introduction:**

Following a preliminary work validating the technological feasibility of an adaptive workflow with Ethos for whole-breast cancer, this study aims to clinically evaluate the automatic segmentation generated by Ethos.

**Material and methods:**

Twenty patients initially treated on a TrueBeam accelerator for different breast cancer indications (right/left, lumpectomy/mastectomy) were replanned using the Ethos^®^ emulator. The adaptive workflow was performed using 5 randomly selected extended CBCTs per patient. The contours generated by artificial intelligence (AI) included both breasts, the heart, and the lungs. The target volumes, specifically the tumor bed (CTV_Boost), internal mammary chain (CTV_IMC), and clavicular nodes (CTV_Nodes), were generated through rigid propagation. The CTV_Breast corresponds to the ipsilateral breast, excluding 5mm from the skin. Two radiation oncologists independently repeated the workflow and qualitatively assessed the accuracy of the contours using a scoring system from 3 (contour to be redone) to 0 (no correction needed). Quantitative evaluation was carried out using the Dice Similarity Coefficient (DSC), Hausdorff Distance (HD), surface Dice (sDSC) and the Added Path Length (APL). The interobserver variability (IOV) between the two observers was also assessed and served as a reference. Lastly, the dosimetric impact of contour correction was evaluated. The physician-validated contours were transferred onto the plans automatically generated by Ethos in adaptive mode. The dose prescription was 52.2Gy in 18 fractions for the boost, 42.3Gy for the breast, IMC, and nodes. The CTV/PTV margin was 2mm for all volumes, except for the IMC (5mm). Dose coverage (D_98%_) was assessed for the CTVs, while specific parameters for organs at risk (OAR) were evaluated: mean dose and V_17Gy_ (relative volume receiving at least 17Gy) for the ipsilateral lung, mean dose and D_2cc_ (dose received by 2cc volume) for the heart, the contralateral lung and breast.

**Results:**

The qualitative analysis showed that no correction or only minor corrections were needed for 98.6% of AI-generated contours and 86.7% of the target volumes. Regarding the quantitative analysis, Ethos’ contour generation outperformed inter-observer variability for all structures in terms of DSC, HD, sDSC and APL. Target volume coverage was achieved for 97.9%, 96.3%, 94.2% and 68.8% of the breast, IMC, nodes and boost CTVs, respectively. As for OARs, no significant differences in dosimetric parameters were observed.

**Conclusion:**

This study shows high accuracy of segmentation performed by Ethos for breast cancer, except for the CTV_Boost. Contouring practices for adaptive sessions were revised following this study to improve outcomes.

## 1 Introduction

Breast cancer (BC) is the most frequently diagnosed cancer and the leading cause of cancer-related death in women worldwide, according to the latest GLOBOCAN study (1). Breast-conserving surgery followed by whole breast irradiation is the current standard of care for patients with early stage BC (2). In recent years, advanced radiotherapy techniques such as Intensity Modulated Radiation Therapy (IMRT), Simultaneous Integrated Boost irradiation or deep-inspiration breath hold have played a crucial role in improving the precision of radiation delivery to tumors. These techniques effectively maximize the target dose while minimizing toxicity to normal tissues and sparing surrounding organs at risk (OAR) (3–6).

Adaptive radiotherapy (ART) is also being investigated for this indication (7). In fact, breast cancer patients have many anatomical variations: heart movement, breathing, arm position can all lead to changes in breast position and shape, as can seroma and swelling following radiation or surgery (8). The ART would therefore allow treatment margins to be reduced, and consequently the volume irradiated. Nevertheless, segmentation remains a key step in online ART (oART), as it must be as short as possible while ensuring high delineation accuracy, given the significant consequences of radiation treatment errors.

With the advent of artificial intelligence (AI) and deep learning (DL), the accuracy of auto-segmentation has improved significantly, particularly for organs at risk, and its use in routine clinical practice is now widespread (9). Various commercial software solutions are available for auto-segmentation and offer high-quality, consistent contours with comparable performance (10, 11). However, delineation of the clinical target volume (CTV) is more challenging due to factors such as physician experience, contouring techniques, and variations in delineation guidelines (12–14). As a result, manual peer review and quality assurance procedures are still recommended before clinical application (15, 16).

The Varian Ethos system (Varian Medical Systems, Palo Alto, CA, USA) uses Cone Beam Computed Tomography (CBCT)for oART and employs DL-based algorithm and structure-guided deformation for structure segmentation (17). So far, Ethos’ experience with oART for breast cancer has been limited to partial breast irradiation (18, 19) and our own technological feasibility study for whole-breast irradiation (20). To our knowledge, this is the first clinical study to evaluate the performance of Ethos auto-segmentation in breast cancer with regional lymph nodes.

## 2 Material and methods

### 2.1 Patient selection

This study was conducted using data from 20 patients who were treated for invasive breast cancer between November 2021 and December 2022 on a TrueBeam accelerator. Patients were included regardless of age, histological subtype, tumor grade, type of surgery (lumpectomy or mastectomy), or whether they received neoadjuvant chemotherapy. Patient characteristics are summarized in **Table 1**.

**Table 1:** Patient characteristics, including treatment side, type of surgery, and volume of breast/chest wall CTV.

Patient	Laterality	Type	CTV Breast/Chest Wall Volume (cc)
1	Right	Conserving surgery	802.5
2	Right	Conserving surgery	489.7
3	Right	Conserving surgery	904.6
4	Right	Conserving surgery	501.2
5	Right	Conserving surgery	381.5
6	Left	Conserving surgery	575.0
7	Left	Conserving surgery	754.2
8	Left	Conserving surgery	692.5
9	Left	Conserving surgery	870.3
10	Left	Conserving surgery	475.4
11	Right	Mastectomy	415.9
12	Right	Mastectomy	274.0
13	Right	Mastectomy	527.8
14	Right	Mastectomy	250.3
15	Right	Mastectomy	516.1
16	Left	Mastectomy	384.5
17	Left	Mastectomy	237.6
18	Left	Mastectomy	368.5
19	Left	Mastectomy	621.1
20	Left	Mastectomy	623.6

### 2.2 Treatment planning

Patients underwent Computed Tomography (CT) scans (GE Optima CT580, General Electric Healthcare, Waukesha, WI, USA) with a 2.5 mm slice thickness, in the supine position, free breathing, with both arms positioned above the head and supported by a personalized foam cushion.

The ESTRO consensus guidelines (21, 22) were used to delineate target volumes, breast/wall, and axillary (Berg I); subclavicular (Berg II, III) and supraclavicular (Berg IV) lymph nodes (Nodes hereafter); and internal mammary chain (IMC). Organs at risk were delineated following French RecoRad 2022 (23) recommendations using TheraPanacea software (24).

The prescribed doses for target volumes were 52.2 Gy for the tumor bed (boost) and 42.3 Gy for the breast, internal mammary chain (CTV_IMC), and clavicular lymph nodes (CTV_Nodes) over 18 fractions. CTV-PTV margins were set at 2 mm for all areas except for the IMC, where a 5 mm margin was applied. Dose constraints for the CTVs and organs at risk (OAR) are outlined in **Table 2**. The dose prescription, PTV margins, and dose constraints were based on the clinical trial “Adaptive radiotherapy in hypersensitive and high locoregional risk breast cancer (SAHARA-04).” (25).

**Table 2:** Dose constraints for CTVs and organs at risk.

CTV constraints			
CTV Boost	D_98%_ ≥ 49.6Gy	D_2%_ ≤ 108%
CTV Breast/Chestwall	D_98%_ ≥ 40.2Gy	D_2%_ ≤ 108%
CTV nodes (IMC and CLN)	D_98%_ ≥ 38.07Gy	D_2%_ ≤ 106%
OAR constraints			
Heart	V_17Gy_ < 10%	V_35Gy_ < 5%
Ipsilateral lung	V_17Gy_ < 30%	D_mean_ < 16Gy
Lungs	V_17Gy_ < 22%	
Brachial plexus	D_max_ < 46.25Gy	
Spinal cord	D_max_ < 38.54Gy	
Contralateral breast	D_mean_ < 2Gy	
LAD coronary	D_max_ < 17Gy (if possible)	

### 2.3 Ethos auto-segmentation

The Ethos adaptive workflow for breast cancer was reproduced using a Varian Ethos emulator (v1.1, Varian Medical Systems, Palo Alto, CA). The AI generates the contours of the influencer structures (also known as organs that influence on the shape and position of the target), namely the right and left breasts (or chest walls), both lungs and the heart. The Varian’s in house AI-based algorithm uses convolutional neural networks to create the influencers (17). The contours of the target volumes are then generated by elastic or rigid registration, according to the user’s choice, to define the CTV_Boost, the CTV_IMC and the CTV_Nodes. The target propagation is based on structure-guided deformations (resulting from the influencer and bone structures generated in the previous step). Our previous study showed a better contour accuracy with rigid propagation and will be the reference propagation for this study (20). The CTV_Breast and CTV_Chestwall are derived structures from the breast and chestwall excluding the 5mm beneath the skin.

### 2.4 Contour accuracy

For each patient, 5 extended CBCTs performed initially for their treatment, were randomly selected in order to simulate 5 adaptive sessions. First, two radiation oncologists independently repeated the adaptive workflow and each Ethos’ auto-segmentation contour (influencers and target volumes) were reviewed. A qualitative evaluation using a physician’s rating was performed for each structure and each adaptive session (i.e 8 structures per CBCT and a total of 100 CBCTs). The scores were defined as follows:

0- No correction needed

1- Minor corrections (≤ 25% of the structure volume)

2-Major corrections (>25% of the structure volume)

3- Contour not usable

Once the score was assigned, the radiation oncologists corrected the contours online, if necessary, by comparing them with the original contours delineated on the simulation CT. A quantitative study was then carried out by comparing the automatically generated contours and the physicians’ contours using similarity metrics. The Dice similarity coefficient (DSC) and the Hausdorff distance (HD) were chosen because of their frequent use in the literature (26, 27) and their complementary properties. However, these two metrics do not correlate with the time required to edit contours. The surface Dice similarity coefficient (sDSC) (28) and the added path length (APL) (29) are more suitable for evaluating time savings (29). Unlike the DSC, which measures on the overlap between two volumes, the sDSC measures the similarity between two surfaces. The APL, on the other hand, is defined as the additional distance to overlap the two contours. The interobserver variability (IOV) between the two physicians was also assessed and served as a reference.

### 2.5 Dosimetric evaluation

The automated planning process resulted in the adapted plan generated by Ethos without editing contours. When evaluating the resulting dose to the physicians’ contours, several dose-volume histogram parameters were analyzed using some dose constraints provided by the SAHARA-04 protocol and some additional relevant parameters. To study the target volume coverage (boost, breast/chestwall, IMC and CLN), the dose received by 98% of the volume (D_98%_) was used. For the organs at risk (OAR), the mean dose and the doses received by 2cc (D_2cc_) was recorded for the heart, contralateral breast and both lungs; the volume receiving 17 Gy (V_17Gy_) for the ipsilateral lung was evaluated too. The Mann-Whitney test was applied to assess the significant differences between the doses received by the Ethos contours and the physician contours.

## 3 Results

### 3.1 Contour accuracy

**Figure 1:**
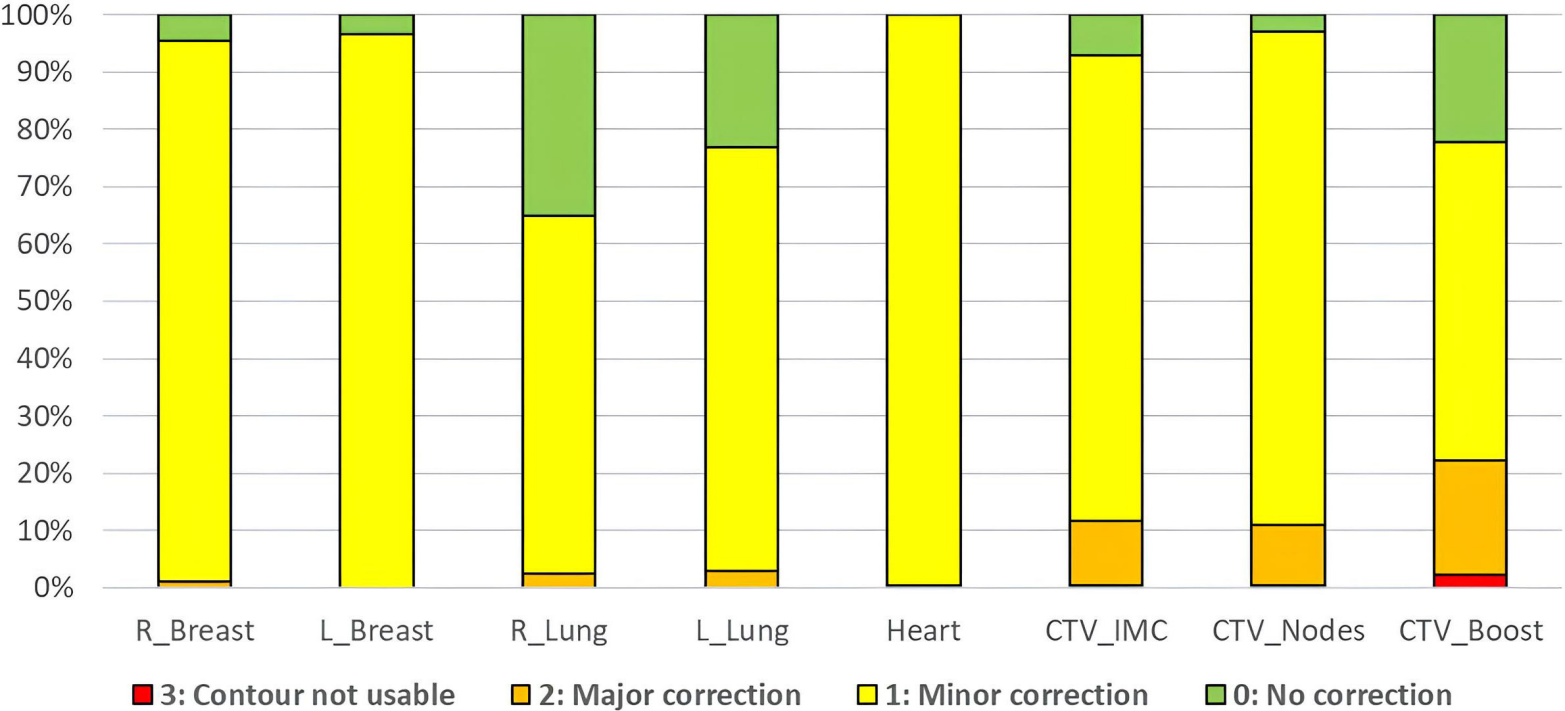
Qualitative scoring by the two radiation oncologists for all structures.

**Figure 1** shows the distribution of qualitative scores assigned by the two radiation oncologists to the structures generated by AI (influencers) and the CTVs produced through rigid propagation. In total, 98.6% of the influencers required no or minor adjustments, compared to 86.7% of the CTV contours. The lowest score was observed for the CTV_Boost with 22.2% of the contours considered not usable or requiring major corrections.

**Figure 2:**
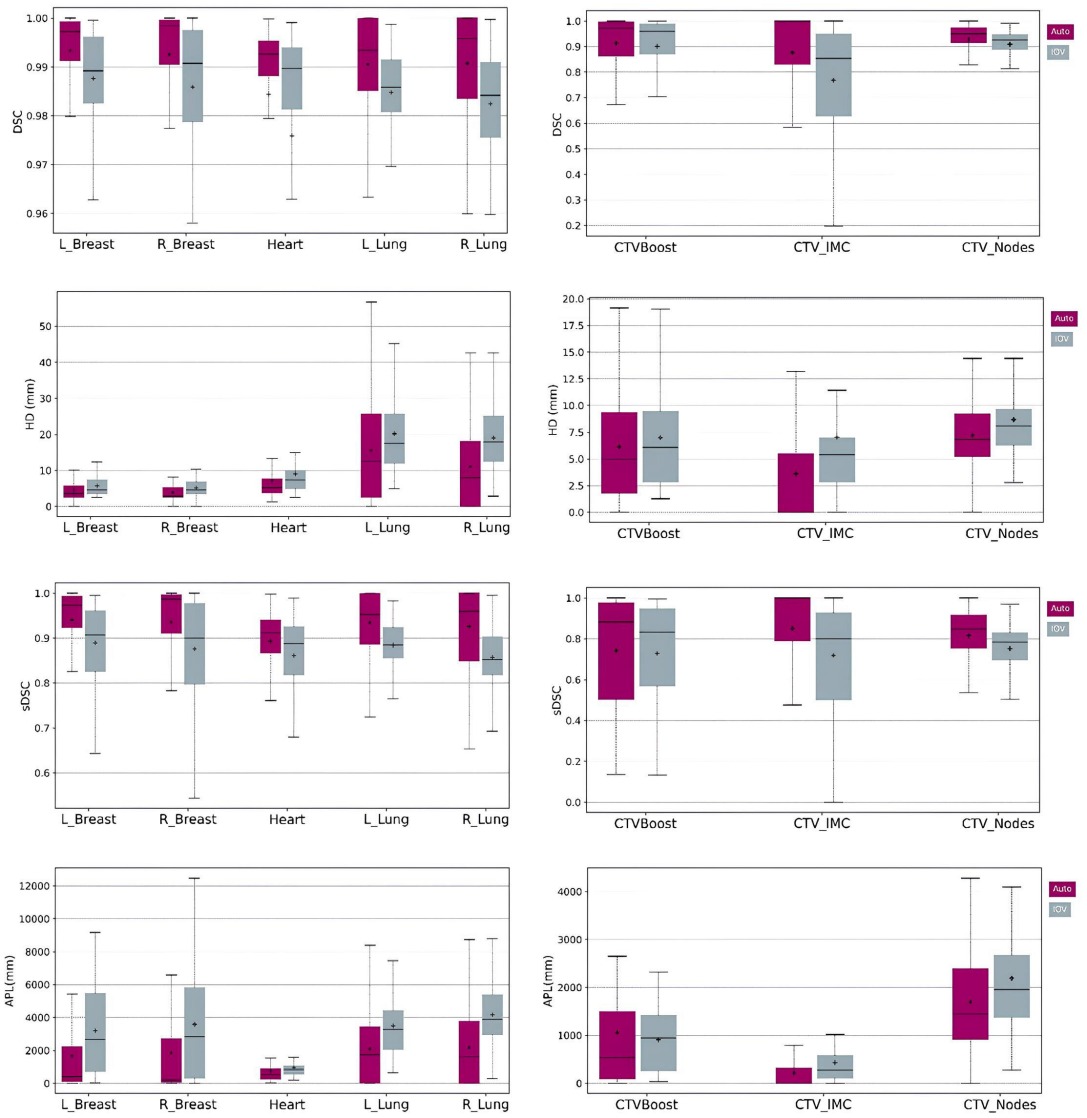
Boxplots of DSC, HD, sDSC and APL for the structures generated by AI (left column) and the structures rigidly propagated (right column). Purple boxplots correspond to the Ethos contours while grey boxplots show the IOV results.

**Figure 3:**
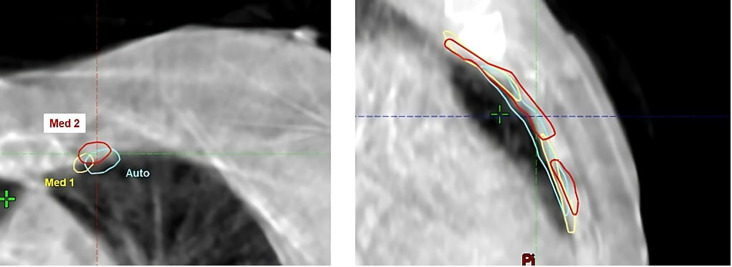
Example of CTV_IMC delineation with the worst DSC results (DSCauto vs Med1 = 0.34; DSCauto vs Med2 = 0.44; DSVIOV = 0.56) in axial (left) and sagittal (right) slices.

Quantitative results, including DSC, HD, sDSC, and APL for the Ethos contours, along with the corresponding inter-observer variability (IOV), are displayed as boxplots in **Figure 2**. For the influencer structures (i.e., both breasts, both lungs, and the heart), the DSC and sDSC median values exceeded 0.9, while the HD median values were below 20mm. The median APLs showed the highest values for both lungs. Additionally, autocontours consistently outperformed inter-observer variability across all metrics. For the CTVs propagated rigidly, the median DSC and sDSC were above 0.8, and the median HD was less than 10mm. The median APLs achieved the highest values for CTV_Nodes. Once again, autocontours outperformed inter-observer variability across all metrics. **Figure 3** shows an example of CTV_IMC delineation comparison with the worst DSC results (DSC_auto vs Med1_ = 0.34; DSC_auto vs Med2_ = 0.44; DSV_IOV_ = 0.56) in axial (left) and sagittal (right) slices.

The comparison of dose metrics for the OARs is presented in **Table 3**. The dose differences between the auto-generated and physician contours were less than 0.1 Gy, except for the heart, where an increase of 0.6 Gy was observed for D_2cc_. However, no statistically significant difference was found.

**Table 3:** Comparison of dose metrics (average ± standard deviation) between auto and physicians’ contours delineated for OARs.

		Auto	Physicians	*p-value*
Ipsi_Lung	D_mean_ (Gy)	8.88 ± 0.92	8.91 ± 0.94	0.355
	V_17Gy_ (%)	15.57 ± 3.06	15.63 ± 3.15	0.437
Contra_Lung	D_mean_ (Gy)	2.49 ± 0.40	2.53 ± 0.41	0.766
Heart	D_mean_ (Gy)	4.08 ± 0.95	4.12 ± 0.95	0.919
	D_2cc_ (Gy)	19.8 ± 6.35	20.36 ± 7.12	0.950
Contra_Breast	D_mean_ (Gy)	2.28 ± 0.43	2.33 ± 0.47	0.910
	D_2cc_ (Gy)	12.3 ± 4.51	12.35 ± 4.48	0.811

**Figure 4:**
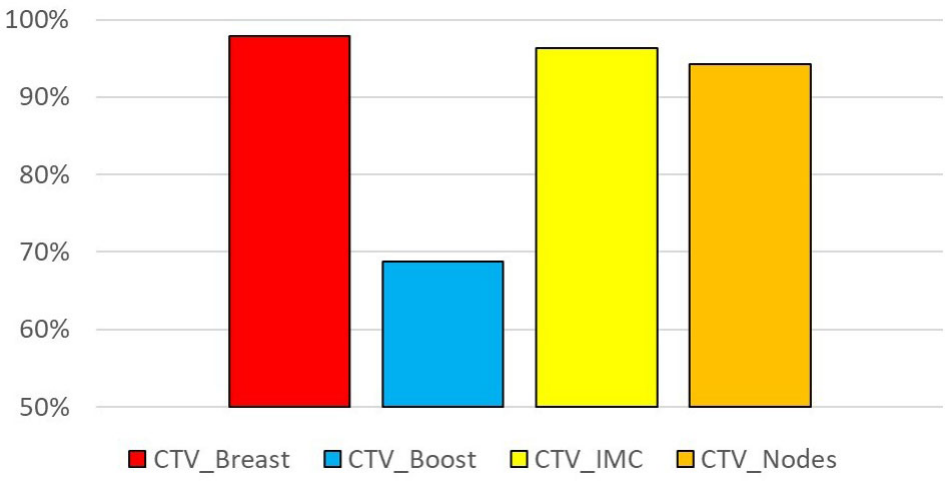
Percentage of sessions fulfilling the coverage constraint: D98% > 95% for CTV_Breast and CTV_Boost, D95% > 90% for CTV_IMC and CTV_Nodes.

**Figure 5:**
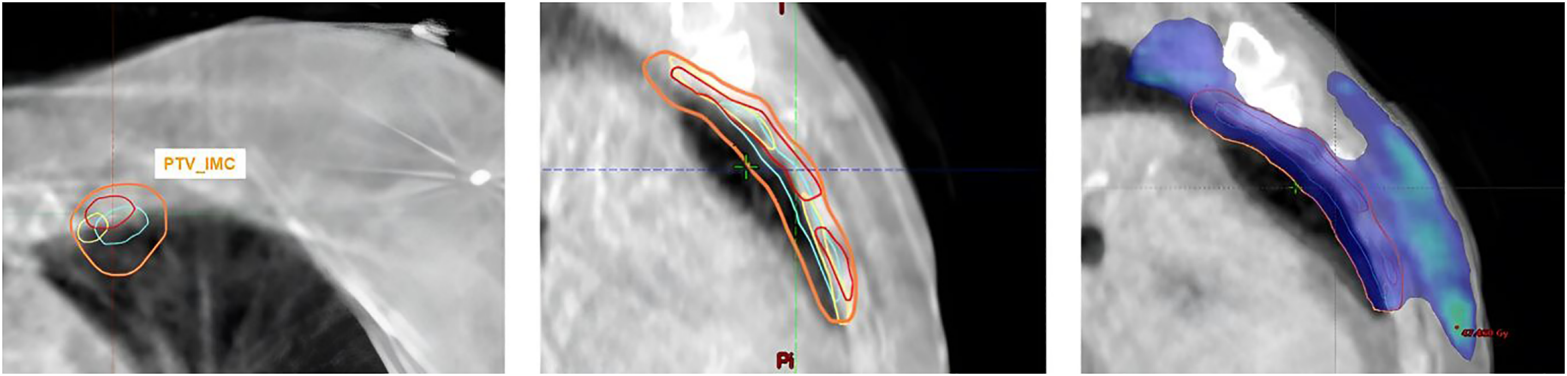
Example of CTV_IMC coverage with the worst DSC results but acceptable CTV dose constraint (D98% Med1 = 96.2%; D98% Med2 = 97%; D98%Auto = 97.4%).

The results of the dosimetric evaluation of the CTVs are shown in **Figure 4**. The CTV coverage constraints, based on D_98%_ > 95% for the CTV_Boost and CTV_Breast, and D_98%_ > 90% for the CTV_IMC and CTV_Nodes, were met for 97.9% of the breast structures, 68.8% of the tumor bed structures, 96.3% of the IMC structures, and 94.2% of the CLN structures. **Figure 5** presents the previous example with the lowest DSC results (**Figure 4**), yet the CTV_IMC coverage still meets the dosimetric constraint (D_98%_ > 90%).

## 4 Discussion

The aim of this study was to evaluate the clinical application of Ethos auto-segmentation for online whole breast ART and the dosimetric impact of physician corrections. While the performance of the Ethos auto-segmentation in the pelvic region is well documented (30–34), there are currently no published studies focusing on its use in whole-breast cancer.

First, the practical relevance was demonstrated by geometric similarity metrics that exceeded inter-observer variation, with most structures needing no or minor adjustment. The Ethos system generated highly accurate influencer structures, with DSC values above 0.9 and HD values below 20 mm. The highest APL was achieved for the lungs due to their large volume, and according to the Vaassen correlation (29), the lungs would require the most correction time. Regarding the structures produced through rigid propagation (i.e CTV), the DSC and HD results are satisfactory with values superior to 0.8 and below 10mm, respectively. CTV_Nodes was the structure that required more time for correction due to the highest APL. Our results are in agreement with the other DL-based segmentation algorithms (35–40). Almberg et al. (35) trained and validated their own DL-segmentation model and achieved median DSC values of 0.96, 0.98 and 0.94 for the heart, both lungs and breast, respectively. For the CTV (IMC and Nodes), their results ranged from 0.70 to 0.81, which were lower than those in our study. This discrepancy is likely due to the different methods used to generate these structures: Ethos uses rigid propagation for CTVs, whereas Almberg et al. employed DL-based segmentation.

Evaluating the dosimetric impact of contour corrections is a key step in assessing system performance, particularly in an online adaptive workflow where time constraints are critical (16). For example, although the lung structure had the highest APL median value, correction were deemed unnecessary due to their minimal dosimetric impact. In contrast, the CTV_IMC showed the largest deviation in similarity metrics relative to its volume, yet dose coverage remained unaffected, largely because a larger PTV margin of 5 mm (compared to 2 mm for other CTVs) was used to account for segmentation uncertainties. The most significant dosimetric impact was observed for CTV_Boost, with only 68.8% of the structures meeting the dose coverage requirement. Despite a standardized protocol for surgical clipping of the breast tumor bed to facilitate accurate localization of the CTV, CTV_Boost delineation in our department is performed manually and is subject to interpretation. In addition, accurate assessment of the tumor bed on CBCT images, as required by the Ethos adaptive workflow, can be challenging due to poor image quality, particularly in soft tissue. Recently, Li et al. (41) developed a contrast learning-based generative model to generate of high-quality synthetic CT from low-quality CBCT and evaluated its performance for post-breast-conserving patients. This method improved the target delineation, such as the tumor bed region. In addition, the newly commercialized CBCT technology, the HyperSight imaging solution (Varian Medical Systems), has shown superior image quality compared to previous imaging on the Ethos platform, along with HU accuracy sufficient for direct dose calculation using the acquired image data (42) and could improve the target delineation in soft tissue (43, 44). However, its performance has not yet been assessed for breast cancer.

As a result of this study, a change in contouring practice for CTV_Boost was implemented. In adaptive treatments, CTV_Boost is now defined by an automatic expansion around the surgical clips, which are more easily visible on CBCT images. Additionally, a procedural guide was created to assist radiation oncologists in managing the breast oART workflow, which includes the following guidelines:

- No correction required for influencer structures (heart, both lungs, and breasts)
- No correction needed for CTV_IMC
- Special attention should be given to the CTV, allowing for rigid movement of the structure (no deformation)
- Verify the surgical clip delineation for CTV_Boost

The primary limitation of this study was the relatively small cohort of 20 patients. Notably, no specific anatomies, such as breast expanders, which could challenge Ethos segmentation performance, were included. Additionally, the study utilized CBCT data from a TrueBeam accelerator instead of the Ethos system as whole-breast treatments were only performed on a C-Arm linac during the study period in our department. However, Cai et al. (45) demonstrated that O-ring CBCT offers equivalent or superior image quality compared to C-Arm CBCT images. Therefore, it is reasonable to assume that the contours generated by Ethos on CBCT images from both the TrueBeam and the Ethos systems are comparable, if not improved.

## 5 Conclusion

This study demonstrated the high accuracy of segmentation performed by Ethos for breast cancer, with the exception of the CTV_Boost. Following this study, contouring practices for adaptive sessions were revised to enhance outcomes and reduce the segmentation workload.

## Data Availability

The original contributions presented in the study are included in the article/supplementary material. Further inquiries can be directed to the corresponding author.
